# Association of epilepsy, anti-epileptic drugs (AEDs), and type 2 diabetes mellitus (T2DM): a population-based cohort retrospective study, impact of AEDs on T2DM-related molecular pathway, and via peroxisome proliferator-activated receptor γ transactivation

**DOI:** 10.3389/fendo.2023.1156952

**Published:** 2023-06-02

**Authors:** Ni Tien, Tien-Yuan Wu, Cheng-Li Lin, Fang-Yi Chu, Charles C. N. Wang, Chung Y. Hsu, Fuu-Jen Tsai, Yi-Jen Fang, Yun-Ping Lim

**Affiliations:** ^1^ Department of Laboratory Medicine, China Medical University Hospital, Taichung, Taiwan; ^2^ Department of Medical Laboratory Science and Biotechnology, China Medical University, Taichung, Taiwan; ^3^ Graduate Institute of Clinical Pharmacy, College of Medicine, Tzu Chi University, Hualien, Taiwan; ^4^ Department of Pharmacy, Taichung Tzu Chi Hospital, Buddhist Tzu Chi Medical Foundation, Taichung, Taiwan; ^5^ Management Office for Health Data, China Medical University Hospital, Taichung, Taiwan; ^6^ School of Chinese Medicine, College of Medicine, China Medical University, Taichung, Taiwan; ^7^ Department of Pharmacy, College of Pharmacy, China Medical University, Taichung, Taiwan; ^8^ Department of Bioinformatics and Medical Engineering, Asia University, Taichung, Taiwan; ^9^ Center for Precision Health Research, Asia University, Taichung, Taiwan; ^10^ Graduate Institute of Biomedical Sciences, China Medical University, Taichung, Taiwan; ^11^ School of Chinese Medicine, College of Chinese Medicine, China Medical University, Taichung, Taiwan; ^12^ Department of Medical Research, China Medical University Hospital, Taichung, Taiwan; ^13^ Division of Medical Genetics, China Medical University Children’s Hospital, Taichung, Taiwan; ^14^ Department of Biotechnology and Bioinformatics, Asia University, Taichung, Taiwan; ^15^ Research Center for Environmental Medicine, Kaohsiung Medical University, Kaohsiung, Taiwan; ^16^ Ph.D. Program in Environmental and Occupational Medicine, College of Medicine, Kaohsiung Medical University and National Health Research Institutes, Kaohsiung, Taiwan; ^17^ Department of Environmental Health, Graduate Institute of Clinical Medicine, Kaohsiung Medical University, Kaohsiung, Taiwan; ^18^ Department of Post-Baccalaureate Medicine, College of Medicine, National Chung-Hsing University, Taichung, Taiwan; ^19^ Digestive Disease Center, Show Chwan Memorial Hospital, Changhua, Taiwan; ^20^ Department of Internal Medicine, China Medical University Hospital, Taichung, Taiwan

**Keywords:** epilepsy, anti-epileptic drugs, type 2 diabetes mellitus, next-generation RNA sequencing, peroxisome proliferator-activated receptor γ

## Abstract

**Introduction:**

A potential association between epilepsy and subsequent type 2 diabetes mellitus (T2DM) has emerged in recent studies. However, the association between epilepsy, anti-epileptic drugs (AEDs), and the risk of T2DM development remains controversial. We aimed to conduct a nationwide, population-based, retrospective, cohort study to evaluate this relationship.

**Methods:**

We extracted data from the Taiwan Longitudinal Generation Tracking Database of patients with new-onset epilepsy and compared it with that of a comparison cohort of patients without epilepsy. A Cox proportional hazards regression model was used to analyze the difference in the risk of developing T2DM between the two cohorts. Next-generation RNA sequencing was used to characterize T2DM-related molecularchanges induced by AEDs and the T2DM-associated pathways they alter. The potential of AEDs to induce peroxisome proliferator-activated receptor γ (PPARγ) transactivation was also evaluated.

**Results:**

After adjusting for comorbidities and confounding factors, the case group (N = 14,089) had a higher risk for T2DM than the control group (N = 14,089) [adjusted hazards ratio (aHR), 1.27]. Patients with epilepsy not treated with AEDs exhibited a significantly higher risk of T2DM (aHR, 1.70) than non-epileptic controls. In those treated with AEDs, the risk of developing T2DM was significantly lower than in those not treated (all aHR ≤ 0.60). However, an increase in the defined daily dose of phenytoin (PHE), but not of valproate (VPA), increased the risk of T2DM development (aHR, 2.28). Functional enrichment analysis of differentially expressed genes showed that compared to PHE, VPA induced multiple beneficial genes associated with glucose homeostasis. Among AEDs, VPA induced the specific transactivation of PPARγ.

**Discussion:**

Our study shows epilepsy increases the risk of T2DM development, however, some AEDs such as VPA might yield a protective effect against it. Thus, screening blood glucose levels in patients with epilepsy is required to explore the specific role and impact of AEDs in the development of T2DM. Future in depth research on the possibility to repurpose VPA for the treatment of T2DM, will offer valuable insight regarding the relationship between epilepsy and T2DM.

## Introduction

Diabetes mellitus (DM) is a chronic metabolic disorder with various causes, characterized by elevated blood glucose levels both in fasting and postprandial states. This condition leads to multiple complications due to deficient insulin secretion or responsiveness, hindering the metabolism of carbohydrates (1). According to the International Diabetes Federation Diabetes Atlas 9^th^ Edition 2021 (http://www.idf.org/), the number of individuals affected by DM worldwide has risen to 537 million, with three out of four adults living in low- and middle-income countries suffering from this condition. Furthermore, almost 12% of global health expenditure is devoted to DM treatment (1). The prevalence of DM is increasing globally and is projected to rise to 643 million by 2030. DM can be classified into three main types based on the underlying causes: [1] type 1 DM (T1DM), resulting from a deficiency in insulin secretion; [2] type 2 DM (T2DM), which accounts for 90–95% of the DM population, caused by insulin resistance and often an inadequate compensatory insulin secretory response; and [3] gestational DM, a type of DM characterized by high blood glucose during pregnancy with an increased risk of T2DM in the newborn (1). Long-term hyperglycemia is associated with complications such as coronary artery disease (CAD), cerebrovascular disease (CVD), renal failure, blindness, limb amputation, neurological defects, and premature death (1). The primary DM treatment is oral hypoglycemic drugs and insulin injections (1, 2). Additionally, T2DM treatment, and to a lesser extent T1DM, involves lifestyle changes. The oral antihyperglycemic agents used include sulfonylureas (which increase insulin secretion), biguanides (insulin sensitizers, e.g., metformin), α-glucosidase inhibitors (which slow the digestion of starch in the small intestine), meglitinides (which increase insulin secretion), dipeptidyl peptidase-4 inhibitors (DPP-4 inhibitors) (which increase insulin secretion), thiazolidinediones (TZDs, agonists of PPARγ), and sodium-glucose cotransporter (SGLT)-2 inhibitors (2).

Epilepsy is a neurological disorder characterized by recurrent seizures resulting from various factors, such as neurobiological, cognitive, psychological, and social factors (3, 4). The prevalence of epilepsy is 5-10 cases per 1000 persons in most countries (3). Recent studies have evaluated the association between epilepsy and comorbidities like DM, celiac disease, thyroid disease, sclerosis, and systemic lupus erythematosus (5–7). Patients with epilepsy have higher mortality rates than the general population, owing primarily to pathological causes such as CVD, stroke, and dementia, all of which are also associated with T2DM (8, 9).

Abnormalities in blood glucose levels can trigger focal motor seizures by disrupting the balance between neuronal inhibition and excitation (10). T2DM is associated with an increased risk of neurological disorders (11). Although limited, the available data suggest an association between T2DM and epilepsy development. A population-based study revealed that severe hypoglycemia is linked to an increased risk of epilepsy in T2DM patients. Additionally, T2DM independently increases the risk of epilepsy development (11). Another review supports the association between T2DM and epilepsy (12). A study showed that the incidence of epilepsy in T2DM patients was 1.5 times higher than that in matched controls (11). Epilepsy often co-occurs with obesity in both children and adults. Insulin resistance and pancreatic islet β-cell dysfunction are two primary mechanisms through which obesity leads to T2DM (13). Adiponectin deficiency is a common feature of these pathologies. Adiponectin stimulates fatty acid (FA) oxidation and glucose uptake in skeletal muscles (14). These factors may contribute to the development of T2DM in people with epilepsy or vice versa.

Anti-epileptic drugs (AEDs) are the most commonly used therapy for managing epilepsy, providing lifelong patient treatment. AEDs can effectively control symptoms in about 75% of patients (15), but they can also have significant metabolic side effects, such as insulin resistance (16). Phenytoin (PHE) is a widely used AED that has been associated with elevated fasting plasma glucose levels and increased insulin resistance in a study of 98 patients undergoing AED monotherapy (17). PHE has also been found to alter insulin secretion and increase blood glucose levels in animal models, potentially contributing to insulin resistance (18, 19). Insulin resistance is linked to higher serum leptin levels, which can induce weight gain (20). In contrast, patients receiving valproate (VPA) treatment for epilepsy may experience endocrine changes that lead to weight gain, but VPA has been shown to stimulate pancreatic β-cells and increase the expression of insulin, leptin, and adiponectin (20). Whether the underlying biological mechanisms of epilepsy and T2DM occur sequentially or concurrently with AED treatment remains to be determined.

Li et al. identified several pathways significantly relevant to T2DM, according to their report (21). They used a text mining tool to extract genes associated with T2DM from a literature database and evaluated the relevant pathways using Fisher’s exact test based on the cumulative hypergeometric distribution. A total of 6,804 genes and 880 canonical pathways associated with T2DM were confirmed based on gene numbers downloaded from the Molecular Signatures Database (MSigDB), which contains an extensive collection of annotated functional gene sets (22). The PolySearch text mining system was used to identify candidate genes associated with T2DM, which can analyze multiple information sources such as PubMed, OMIM, DrugBank, and Swiss-Prot to produce a list of relevant concepts. The top 10 statistically relevant pathways related to T2DM, ranked by P-value, were attributed to five classes of signaling pathways, including adipocytokine, inflammatory, peroxisome proliferators activated receptor (PPAR), insulin, and T2DM pathway (21).

Peroxisome proliferator-activated receptors (PPARs) are members of the nuclear receptor superfamily that consist of three subtypes: PPARα (*NR1C1*), PPARβ/δ (*NR1C2*), and PPARγ (*NR1C3*). These receptors are activated by lipid-derived substrates and play distinct roles in energy metabolism (23). PPARα, the first subtype identified, is mainly expressed in the liver, heart, and brown adipose tissue, where it mediates FA oxidation and is the target of hypolipidemic fibrates. PPARβ/δ shares similar functions with PPARα and is ubiquitously expressed, playing a crucial role in FA oxidation in key metabolic tissues such as skeletal muscle, liver, and heart (23). PPARγ is the most highly expressed subtype in white/brown adipose, endothelial, and muscle tissues, and is a master regulator of adipogenesis, modulating processes such as FA transport, uptake by cells, intracellular binding and activation, lipid catabolism, and FA storage. It also significantly modulates glucose homeostasis and insulin sensitivity (23, 24). Upon activation, PPARs form a heterodimer with the retinoid X receptor (RXR) and bind to a specific DNA cis-acting element called a peroxisome proliferator response element (PPRE), thereby initiating gene transcription (25). Prostaglandin J2 metabolites, oxidized low-density lipoprotein particles, and synthetic TZD compounds such as rosiglitazone and pioglitazone are PPARγ activators (24). These drugs have a high affinity for PPARγ, which is an attractive pharmacological target for treating T2DM since it simultaneously modulates several of the pathways disrupted in T2DM and metabolic syndrome by enhancing the expression of genes involved in glucose and lipid metabolism and increasing the expression of glucose transporter type 4 (GLUT4), an insulin-stimulated glucose transport system expressed in muscle cells and the expression of GLUT4 is highly regulated by PPARγ (2, 26, 27). In mice, muscle-specific deletion of PPARγ resulted in severe insulin resistance with milder defects in adipose tissue and liver, altered adipokine expression, increased adiposity, hyperinsulinemia, glucose intolerance, and hypertriglyceridemia (28). Given that skeletal muscle is a major site of fuel oxidation, the loss of muscle PPARγ could affect and impede the utilization of FA and glucose by skeletal muscle, contributing to the development of insulin resistance and T2DM.

The link between epilepsy and T2DM remains unclear, lacking adequate epidemiological evidence. Our goal was to use the National Health Institute (NHI) database to investigate the correlation between epilepsy, AED type, and the incidence of newly diagnosed T2DM in epileptic patients. We also examined the potential underlying biological mechanisms driving the impact of PHE and VPA on T2DM risk. Additional research is needed to clarify the association between epilepsy and T2DM and whether AEDs are involved in the pathophysiological changes that lead to T2DM. Next-generation sequencing (NGS) can help to identify molecular pathways related to each condition and provide insight into comorbidities by assessing molecular interaction networks. Our novel approach integrated clinical database information with data on high-level molecular changes consistently associated with known comorbid diseases.

Our study found that VPA activates PPARγ and increases glucose content in treated muscle cells, which play a significant role in fuel oxidation. Additionally, it enhances the expression of insulin receptors (IRs) and GLUT4. These results suggest that VPA enhances glucose uptake by upregulating GLUT4 expression and may be used as an insulin-sensitizing agent.

## Materials and methods

### Data sources

The Taiwan government built a database with high coverage, the National Health Insurance Research Database (NHIRD), which included nearly 99% of Taiwan population. The claims data was obtained from Taiwan National Health Research Institute (NHRI), with the authorization from the NHI Administration, Ministry of Health and Welfare (MHW). This study used the Longitudinal Generation Tracking Database 2005 (LGTD 2005), a subsection of the NHIRD. The LGTD includes the mentioned claims data from 1996 to 2017 belonging to 2,000,000 patients randomly selected from the NHIRD, data available for public access. This database includes information on ambulatory care, inpatient care, dental services, prescription drugs, medical institutions, and physician information. To protect the privacy of the patients and to secure their confidentiality, the identification number of each patient was encrypted, precluding the possibility of the ethical violation of the data. The diagnoses in Taiwan NHI are coded according to the International Classification of Diseases, Ninth & Tenth Revision, Clinical Modification (ICD-9-CM & ICD-10-CM). The Research Ethics Committee of China Medical University and Hospital in Taiwan approved the study [CRREC-109-169 (CR-1)].

### Study population

In this study, we included two cohorts of patients: epileptic and non-epileptic (comparison cohort). The epileptic cohort included patients with epilepsy (ICD-9-CM code: 345 or ICD-10-CM code: G40) newly diagnosed from 2000 to 2016. The date when epilepsy was diagnosed was defined as the index date. The non-epileptic comparison cohort included randomly selected patients without epilepsy registered in the LGTD in 2005. It was matched with the epileptic cohort at a 1:1 ratio based on age (per 5 years), sex, and index year. Patients in both cohorts were less than 20 years old, and those with T2DM (ICD-9-CM codes: 250. × 0 and 250. × 2 or ICD-10-CM code: E11) diagnosed before the index date, or with incomplete age or sex information were excluded. The follow-up of this study continued up until the patients withdrawn the insurance, T2DM was diagnosed or until December 31, 2017. The T2DM group included epileptic patients who had ≥ 1 admission or ≥ 2 ambulatory care visits for T2DM within a 365-day period between 2000 and 2017. The patients diagnosed with T1DM (ICD-9-CM: 250. × 1 or 250. × 3 or ICD-10-CM code: E10) between 2000 to 2017 were also excluded.

### Outcome measures, comorbidities, and medications

Comorbidities were defined as comorbidities preceding the index date and included hypertension, stroke, hyperlipidemia, atrial fibrillation (AF), chronic obstructive pulmonary disease (COPD), coronary artery disease (CAD), congestive heart failure (CHF), alcohol-related illness, asthma, obesity, and cancer. We analyzed the impact of the following associated medication used in epileptic patients: steroids, statins, thiazide diuretics, and beta-blockers. We extracted data on AEDs, defined as drugs with ATC classification codes N03A and N05BA09. They were classified into the following therapeutical classes: barbiturates and derivatives [including phenobarbital (PB) and primidone], hydantoin derivatives [including phenytoin (PHE)], benzodiazepines [including clonazepam (CLZ), clorazepate, and diazepam], carboxamide derivatives [including carbamazepine (CBZ) and oxcarbazepine], FA derivatives [including valproate (VPA)], and others [gabapentin (GBP), lamotrigine, levetiracetam, pregabalin, topiramate, and zonisamide]. All these therapeutical agents are available in Taiwan.

### Chemicals, cell cultures, and cytotoxicity assessment

One AED was selected from each group. All chemicals were purchased at the highest-purity grade available and dissolved appropriately. The HepG2, human and mouse rhabdomyosarcoma (RD and C2C12, respectively) cell lines were maintained in Minimum Essential Medium alpha (αMEM: HepG2) and Dulbecco’s modified Eagle’s medium (DMEM: RD, C2C12), respectively. All cultured cells were maintained in a humidified atmosphere at 37°C with 5% CO_2_. Cell viability was measured as previously described by using a modified acid phosphatase (ACP) assay with 4-nitrophenyl phosphate disodium salt as a substrate (29).

### mRNA sequencing by using Illumina HiSeq

RD cells were treated with PHE and VPA to study their effects on T2DM pathway. After a 72-hour exposure to drug treatment, gene expression was evaluated in triplicate. Total RNA of each sample was extracted using TRIzol reagent (Invitrogen)/RNeasy Mini Kit (Qiagen), according to manufacturer’s instruction. Total RNA of each sample was characterized quantitively and qualitatively using an Agilent 2100 Bioanalyzer (Agilent Technologies, Palo Alto, CA, USA), NanoDrop spectrophotometer (Thermo Fisher Scientific Inc.), and 1% agarose gel electrophoresis. Next, 1 μg total RNA with RNA integrity number above 6.5 was used for the library preparation. NGS libraries were prepared according to manufacturer’s protocol. Poly(A) mRNA isolation was performed using poly(A) mRNA Magnetic Isolation Module or rRNA removal Kit. Next, mRNA fragmentation and priming were performed using First-strand Synthesis Reaction Buffer and Random Primers. First-strand cDNA was synthesized using ProtoScript^®^ II Reverse Transcriptase (New England Biolabs, Ipswich, MA), and second-strand cDNA was synthesized using Second-strand Synthesis Enzyme Mix (Thermo Fisher Scientific Inc.). The double-stranded cDNA purified using beads was treated with End-Prep Enzyme Mix (New England Biolabs, Ipswich, MA) to repair both ends, and a dA-tailing was added to one reaction, followed by a T-A ligation to add adaptors to both the ends. The size of adaptor-ligated DNA was selected using beads, and fragments of approximately 420 bp (with the approximate insert size of 300 bp) were recovered. Each sample was then amplified using PCR for 13 cycles and P5 and P7 primers, with both primers carrying sequences that can anneal with the flow cell to perform bridge PCR and P7 primer additionally carrying a six-base index allowing for multiplexing. The PCR products were cleaned using beads, validated using Qsep100 (BiOptic, Taiwan), and quantified using Qubit3.0 fluorometer (Invitrogen, Carlsbad, CA, USA). The libraries with different indices were multiplexed and loaded on an Illumina HiSeq instrument (Illumina, San Diego, CA, USA), according to manufacturer’s instructions. Sequencing was performed using a 2 × 150 bp paired-end (PE) configuration; image analysis and base calling were conducted using HiSeq Control Software (HCS) + OLB + GAPipeline-1.6 (Illumina) on the HiSeq instrument. The sequences were processed and analyzed using GENEWIZ (Azenta Life Sciences, Inc. USA).

### Data analysis

For quality control, to obtain high-quality clean data and to remove technical sequences, including adaptors, PCR primers, or fragments, and sequences with quality of bases lower than 20, the data in FASTQ format was filtered using Cutadapt (V1.9.1). Furthermore, reference genome sequences for mapping and gene model annotation files of relative species were downloaded from the genome websites such as UCSC, NCBI, and ENSEMBL. Hisat2 (v2.0.1) was used to index reference genome sequences. Finally, clean data were aligned to the reference genome by using software Hisat2 (v2.0.1). For expression analysis, first, transcripts in the FASTA format were converted from known gff annotation file and indexed properly. Next, based on a reference gene file, HTSeq (v0.6.1) was used to estimate gene and isoform expression levels from PE clean data. For differential expression analysis, we used DESeq2 Bioconductor package, a model based on the negative binomial distribution, and estimated the dispersion and logarithmic fold changes for data-driven prior distributions; an adjusted P (adjP) value < 0.05 was set to detect differentially expressed genes (DEGs). GOSeq (v1.34.1) was used to identify Gene Ontology (GO) terms annotating a list of enriched genes with a significant adjP of < 0.05, and top GO terms were used to plot directed acyclic graph. We used Kyoto Encyclopedia of Genes and Genomes (KEGG) for the systematic analysis of gene functions. This is a collection of databases dealing with genomes, biological pathways, diseases, drugs, and chemical substances (30) (http://en.wikipedia.org/wiki/KEGG). We used in-house scripts to enrich significant DEGs in the KEGG pathways.

### Plasmid construction, transfection, and reporter assay

To study the activation of PPARγ in a luciferase reporter assay, PPARγ expression vectors were amplified from full-length human PPARγ (MGC: 5041, IMAGE:3447380; pCMV-SPORT6; Open Biosystems Inc.) by using PCR and the forward and reverse primers 5′-ACG TTG GTA CAG CTG AAT CCA GAG TCC GCT GA-3′ and 5′-TCT AGA CTA GTA CAA GTC CTT GTA GAT CTC CTG CAG GAG C-3′, incorporating *Kpn*I and *Xba*I sites at the 5′ and 3′ ends, respectively. The PPARγ fragment was then ligated to the pcDNA3 *Kpn*I/*Xba*I site, forming a full-length construct (pcDNA3-PPARγ). To study the activation of the full-length PPARγ receptor, a 3 × repeat of the PPRE 5′-GGA CCA GGA CAA AGG TCA CGT TC-3′ was cloned into a pGL3-basic vector (Promega, Inc.) (31, 32). HepG2 cells were simultaneously co-transfected with the PPARγ reporter system and a β-galactosidase plasmid in each well. Transfections were allowed to proceed for 6 h before drug exposure. After 20 h of drug treatment, the reporter assay was performed as previously described (29).

### Small interfering RNAs and siRNA transfection, T0070907 treatment, RNA isolation, qRT-PCR analysis, western blot analysis, and glucose content assessment assay

Small interfering control RNAs (siControl) and siRNA validated for PPARγ were purchased from Thermo Fisher Scientific (Thermo Fisher Scientific Inc.) and applied to RD cells by using Lipofectamine^®^ RNAiMAX reagent (Thermo Fisher Scientific Inc.) for 48 h. The culture was further incubated with or without VPA for 24 h. The PPARγ antagonist, T0070907 (T007), was added 20 minutes (min) before the 24-hour exposure to the VPA treatment.

After 24-hour treatment with AEDs, total RNA was extracted from C2C12 and RD by using a Direct-zol™ RNA MiniPrep kit (ZYMO Research, Irvine, CA, USA), according to manufacturer’s protocol. It was further subjected to the synthesis of the first-strand cDNA by using a MultiScribe™ reverse transcriptase kit (Thermo Fisher Scientific Inc.). Genes related to glucose homeostasis and PPARγ target genes were analyzed using qRT-PCR and the Luminaris Color HiGreen qPCR master mix (Thermo Fisher Scientific Inc.) in a real-time PCR system under standard procedures. Each pair of specific primers used for real-time PCR analysis were as follows: *mGLUT4* Forward, 5’- GGA AGG AAA AGG GCT ATG CTG-3’, *mGLUT4* Reverse, 5’- TGA GGA ACC GTC CAA GAA TGA-3’; *hGLUT4* Forward, 5’- CAT TCC TTG GTT CAT CGT G-3’, *hGLUT4* Reverse, 5’- ATA GCC TCC GCA ACA TAC-3’; *hIR* Forward, 5’- TTT TCG TCC CCA GGC CAT C-3’, *hIR* Reverse, 5’- GTC ACA TTC CCA ACA TCG CC-3’; *hPPARγ* Forward, 5’- AAA GAA GCC GAC ACT AAA CC-3’, *hPPARγ* Reverse, 5- CTT CCA TTA CGG AGA GAT CC-3’; *β-actin* Forward, 5′-CCT GGC ACC CAG CAC AAT-3′, *β-actin* Reverse, 5′-GCC GAT CCA CAC GGA GTA CT-3′. Cells were treated with AEDs for 24 h, and the expression level of the above-mentioned proteins was measured using Western blot as previously described (29). Glucose content was measured using a commercial colorimetric kit available from BioVision (BioVision, Inc.).

### Molecular docking

Molecular docking techniques are used to predict and investigate the steric and electrostatic complementarity between the PPARγ ligand-binding domain (LBD) and putative ligands. Currently, it is a frequently used tool in drug discovery by predicting the intermolecular framework formed between a protein and a ligand responsible for the modulation of the protein (33). In this study, PPARγ structure was extracted from Protein Data Bank (PDB; 6ILQ) and used as a template to dock VPA on PPARγ LBD by using Discovery Studio 4.5. All crystallized H_2_O molecules were removed from the protein and the substrate, and hydrogen was added into the CDOCKER module. CDOCKER is a CHARMm-based docking method that has been used to generate highly accurate docked poses. The ligands were conceded to tilt around the rigid receptor (34).

### Statistical analysis

To compare the differences between the two cohorts, we used two-sample t-test, when considering age as continuous variable and chi-square test when considering sex, medication, and comorbidities as category variable. The incidence density rate of T2DM was calculated for both cohorts and estimated by dividing the number of events (representing occurrence of T2DM) by follow-up time (per 1000 person-years). The Kaplan-Meier method was used to measure cumulative incidence curves for each cohort and log rank test was applied to assess the difference between two survival curves. We estimated hazard ratios (HRs) and 95% confidence intervals (CIs) for risk of development of T2DM in epileptic and non-epileptic cohorts by using crude and adjusted Cox proportional hazard models. Besides, we evaluated the effect and estimated the risk of T2DM determined by cumulative doses of PHE and VPA, taking into consideration the usage of steroids, thiazide diuretics, and statins. We estimated the annual mean defined daily dose (DDD) of these drugs and further partitioned into three levels. All statistical analyses were performed using SAS statistical software, version 9.4 (SAS Institute Inc., Cary, NC). The figure representing the cumulative incidence curve was plotted by R software. P values < 0.05 were set as having statistical significance.

For the experimental part, the data is presented as mean ± standard error (SE). All experiments were performed at least in triplicate and their mean was used for further analysis by SPSS for Windows, version 20.0 (IBM SPSS, Armonk, NY, USA). One-way analysis of variance (ANOVA) was performed and the significant differences were evaluated by *post hoc* LSD. Results were considered statistically significant at P < 0.05.

## Results

### Baseline characteristics: demographic and association findings

**Figure 1:**
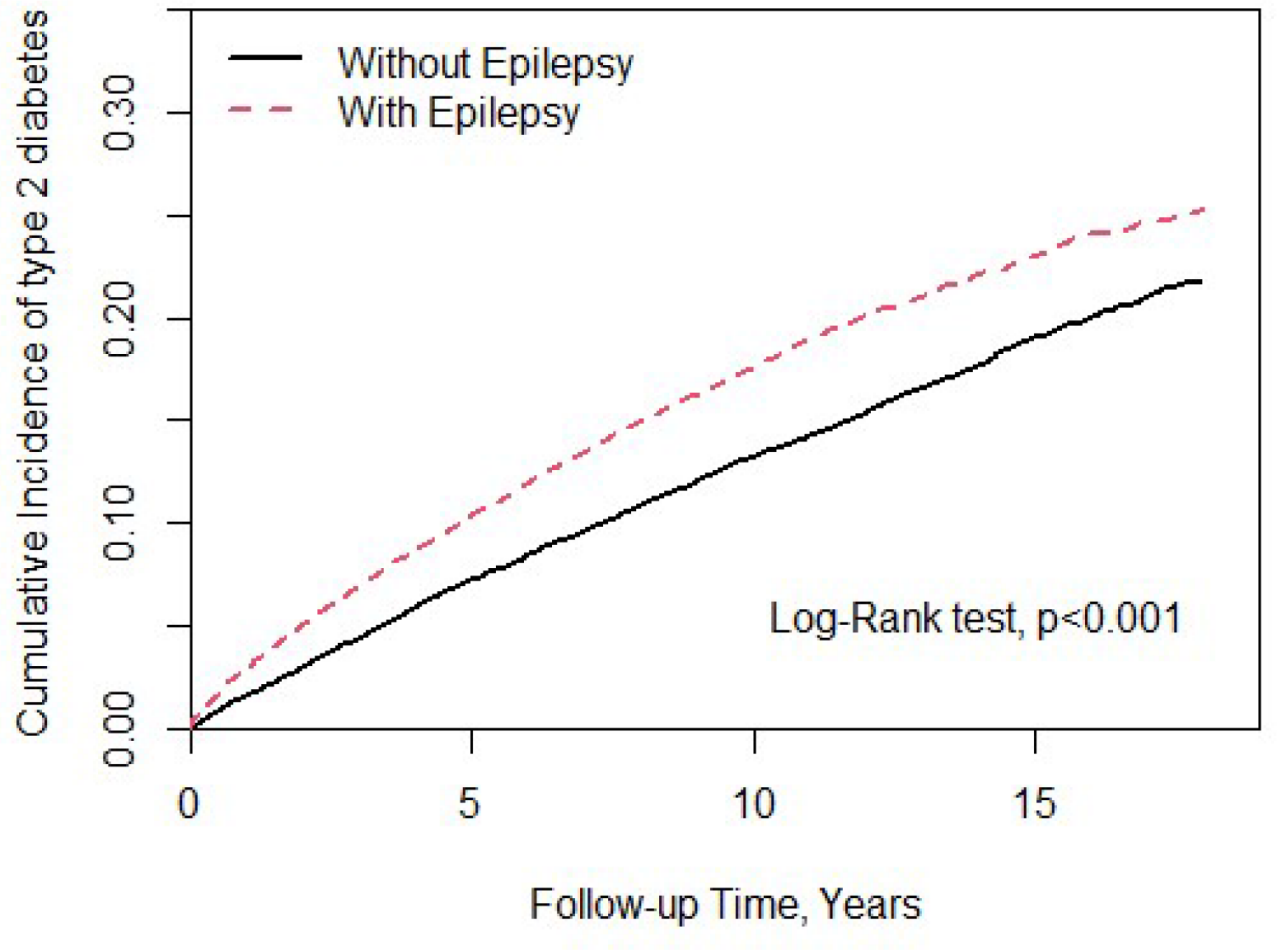
Cumulative incidence of type 2 diabetes mellitus (T2DM) compared between with and without epilepsy using the Kaplan-Meier method.

In this study, we totally enrolled 28,178 patients (**Table 1**), 14,089 patients with epilepsy and 14,089 patients without. In the study population, 61.1% were male. The mean ages of the epileptic cohort and non-epileptic cohort were 49.5 ± 18.9 and 49.0 ± 19.0 years, respectively. In the epileptic cohort, we found a significantly higher proportion of all comorbidities and medications usage than in the non-epileptic cohort. **Figure 1** showed that epileptic patients had significantly higher T2DM cumulative incidence than the non-epileptic cohort (log-rank test P < 0.001).

**Table 1:** Demographic characteristics, comorbidities, and medications in patient with and without epilepsy.

Variable	Epilepsy		P value
	No	Yes	
	N =14089	N =14089	
Sex	N (%)	N (%)	0.99
Female	5485 (38.9)	5485 (38.9)	
Male	8604 (61.1)	8604 (61.1)	
Age, mean (SD)^#^	49.0 (19.0)	49.5 (18.9)	0.02
Stratify age			0.03
≤ 49	7945 (56.4)	7790 (55.3)	
50-65	3043 (21.6)	3009 (21.4)	
≥ 65	3101 (22.0)	3290 (23.4)	
Comorbidity			
Hypertension	3162 (22.4)	5117 (36.3)	< 0.001
Hyperlipidemia	1800 (12.8)	2239 (15.9)	< 0.001
Atrial fibrillation	149 (1.06)	511 (3.63)	< 0.001
Stroke	317 (2.25)	2948 (20.9)	< 0.001
Chronic obstructive pulmonary disease (COPD)	1418 (10.1)	2486 (17.6)	< 0.001
Coronary artery disease (CAD)	1593 (11.3)	2460 (17.5)	< 0.001
Congestive heart failure (CHF)	386 (2.74)	775 (5.50)	< 0.001
Alcohol-related illness	249 (1.77)	1187 (8.43)	< 0.001
Asthma	952 (6.76)	1337 (9.49)	< 0.001
Obesity	78 (0.55)	137 (0.97)	< 0.001
Cancer	303 (2.15)	678 (4.81)	< 0.001
Medication			
Steroids	7236 (51.4)	7771 (55.2)	< 0.001
Statins	962 (6.83)	1348 (9.57)	< 0.001
Diuretics	2683 (19.0)	4728 (33.6)	< 0.001
Beta-blockers	3337 (23.7)	6021 (42.7)	< 0.001

^#^Chi-Square test.

Throughout the study, there were 2,246 and 1,933 patients newly diagnosed with T2DM in the epileptic group and non-epileptic group, respectively (**Table 2**). The incidence of T2DM was 18.6 per 1,000 person-years in the epileptic group and 14.1 per 1,000 person-years in the non-epileptic group. The epileptic patients were 1.31 times more likely to develop T2DM than non-epileptic patients (HR = 1.31, 95% CI = 1.23-1.39). After adjusting for age, sex, comorbidities, and medication, there was still a 1.27-fold increase in the risk of T2DM for the epileptic vs. control group (aHR = 1.27, 95% CI = 1.19-1.36). The sex-specific analysis of the relative risk of T2DM between the epileptic and non-epileptic cohort demonstrated the risk was significantly higher in both women and men with epilepsy than those without. The age-specific analysis indicated the relative risk of T2DM was higher for all age groups in the epileptic cohort than in the non-epileptic cohort. When we assessed the influence of medication on the T2DM risk, the epileptic patients had a significantly increased risk of T2DM when compared with individuals without epilepsy, for both users and non-users of steroids, statins, diuretics, and beta-blockers.

**Table 2:** Comparison of incidence and hazard ratio (HR) of type 2 diabetes mellitus (T2DM) stratified by sex, age, and comorbidities between with and without epilepsy.

	Epilepsy							
	No			Yes				
Variable	Event	PY	Rate^#^	Event	PY	Rate^#^	Crude HR (95% CI)	Adjusted HR^†^ (95% CI)
All	1933	136976	14.1	2246	120807	18.6	1.31 (1.23, 1.39)***	1.27 (1.19, 1.36)***
Sex								
Female	736	53627	13.7	953	47699	20.0	1.45 (1.31, 1.59)***	1.43 (1.30, 1.59)***
Male	1197	83349	14.4	1293	73108	17.7	1.22 (1.13, 1.32)***	1.18 (1.08, 1.28)***
Stratify age								
≤ 49	727	90665	8.02	932	82998	11.2	1.41 (1.28, 1.55)***	1.28 (1.16, 1.42)***
50-64	615	25512	24.1	666	21071	31.6	1.31 (1.17, 1.46)***	1.18 (1.04, 1.32)**
≥ 65	591	20799	28.4	648	16738	38.7	1.32 (1.18, 1.48)***	1.25 (1.11, 1.41)***
Comorbidity^‡^								
No	976	103274	9.45	763	71290	10.7	1.13 (1.03, 1.24)*	1.31 (1.19, 1.45)***
Yes	957	33702	28.4	1483	49517	30.0	1.05 (0.97, 1.14)	1.14 (1.04, 1.25)**
Medication								
Steroids								
No	1064	871444	13.1	1149	71750	16.0	1.23 (1.13, 1.33)***	1.23 (1.12, 1.34)***
Yes	869	55532	15.7	1097	49057	22.4	1.40 (1.28, 1.53)***	1.32 (1.20, 1.45)***
Diuretics								
No	1407	120674	11.7	1417	95957	14.8	1.27 (1.18, 1.37)***	1.32 (1.22, 1.42)***
Yes	526	16302	32.3	829	24851	33.4	1.02 (0.92, 1.14)	1.09 (0.97, 1.23)
Statins								
No	1734	132114	13.1	2006	114613	17.5	1.33 (1.25, 1.42)***	1.31 (1.22, 1.40)***
Yes	199	4862	40.9	240	6194	38.8	0.94 (0.78, 1.14)	0.93 (0.76, 1.15)
Beta-blockers								
No	1326	115246	11.5	1206	84284	14.3	1.25 (1.15, 1.35)***	1.32 (1.21, 1.43)***
Yes	607	21730	27.9	1040	36524	28.5	1.02 (0.92, 1.12)	1.12 (1.00, 1.24)*

Rate^#^, incidence rate, per 1,000 person-years; Crude HR, crude hazard ratio.

Adjusted HR^†^: multivariable analysis including age, sex, and comorbidities of hypertension, hyperlipidemia, stroke, COPD, CAD, CHF, alcohol-related illness, asthma, obesity, cancer, impaired glucose tolerance, and gestational diabetes, and medication of steroids, diuretics, statins and beta-blockers.

Comorbidity^‡^: Patients with any one of the comorbidities, hypertension, hyperlipidemia, stroke, COPD, CAD, CHF, alcohol-related illness, asthma, obesity, cancer, impaired glucose tolerance, and gestational diabetes were classified as the comorbidity group.

*P < 0.05, **P < 0.01, ***P < 0.001.

When considering the patients without epilepsy as reference, epileptic patients without AEDs treatment (aHR = 1.70, 95% CI = 1.54-1.88), with hydantoin derivatives-based monotherapy (aHR = 1.26, 95% CI = 1.16-1.37) and overall epileptic patients receiving treatment with AEDs (aHR = 1.19, 95% CI = 1.11-1.27) had higher risk of T2DM (**Table 3**). Compared to epileptic patients without AEDs treatment, epileptic patients receiving treatment with AEDs had lower risk of T2DM.

**Table 3:** Incidence, crude, and adjusted hazard ratio (HR) of type 2 diabetes mellitus (T2DM) compared among epilepsy patients with and without anti-epileptic drugs (AEDs) treatment compared to non-epilepsy controls.

Variables					Crude HR (95% CI)	Adjusted HR^†^(95% CI)	Adjusted HR^†^(95% CI)
	N	Event	PY	Rate^#^			
Non-epilepsy controls	14089	1933	136976	14.1	1 (Reference)	1 (Reference)	
Epilepsy without AEDs treatment	2399	528	16765	31.5	2.12 (1.93, 2.34)***	1.70 (1.54, 1.88)***	1 (Reference)
Only barbiturate and derivatives	826	122	10793	11.3	0.83 (0.69, 1.00)*	1.01 (0.84, 1.22)	0.60 (0.49, 0.73)***
Only hydantoin derivatives	4961	809	47558	17.0	1.21 (1.12, 1.32)***	1.26 (1.16, 1.37)***	0.74 (0.66, 0.83)***
Only benzodiazepam derivatives	3052	485	28635	16.9	1.20 (1.08, 1.32)***	1.13 (1.02, 1.25)*	0.67 (0.59, 0.76)***
Only carboxamide derivatives	823	120	8011	15.0	1.07 (0.89, 1.28)	1.23 (1.02, 1.48)*	0.72 (0.59, 0.87)***
Only fatty acid derivatives	910	113	5989	18.9	1.29 (1.06, 1.56)**	1.13 (0.93, 1.37)	0.66 (0.54, 0.81)***
Only others^§^	584	69	3056	22.6	1.50 (1.18, 1.91)***	1.24 (0.97, 1.59)	0.73 (0.57, 0.94)***
Overall epilepsy with AEDs treatment	11156	1718	104042	16.5	1.17 (1.10, 1.25)***	1.19 (1.11, 1.27)***	

Rate^#^, incidence rate, per 1,000 person-years; Crude HR, crude hazard ratio.

Adjusted HR^†^: multivariable analysis including age, sex, and comorbidities of hypertension, hyperlipidemia, stroke, COPD, CAD, CHF, alcohol-related illness, asthma, obesity, cancer, impaired glucose tolerance, and gestational diabetes, and medication of steroids, diuretics, statins and beta-blockers.

^§^Other AEDs including gabapentin, lamotrigine, levetiracetam, pregabalin, topiramate, and zonisamide.

*P < 0.05, **P < 0.01, ***P < 0.001.

**Table 4** presents the dose-response relationship between PHE and VPA use and T2DM risk in patients with epilepsy, compared to those without. There was a significantly higher risk of T2DM for patients receiving PHE when using > 150 DDD (aHR = 2.28, 95% CI = 1.99-2.61) than control cohort. However, in epileptic patients treated with VPA, the risk of T2DM decreased dose-dependently with the increase of DDD, although this trend did not reach statistically significance.

**Table 4:** Hazard ratio (HR) and 95% confidence intervals (CIs) for type 2 diabetes mellitus (T2DM) associated with average defined daily doses (DDD) of phenytoin (PHE) and valproate (VPA).

	Crude HR	(95% CI)	Adjusted HR^†^	(95% CI)
Non-epilepsy controls	1.00	(reference)	1.00	(reference)
Epilepsy with PHE				
≤ 30 DDD	0.96	(0.86, 1.07)	1.04	(0.93, 1.16)
31-150 DDD	1.01	(0.88, 1.16)	1.08	(0.94, 1.24)
> 150 DDD	2.19	(1.92, 2.49)***	2.28	(1.99, 2.61)***
P for trend		< 0.001		< 0.001
Epilepsy with VPA				
≤ 40 DDD	0.88	(0.68, 1.13)	1.11	(0.86, 1.44)
41-160 DDD	0.80	(0.55, 1.17)	0.95	(0.65, 1.39)
> 160 DDD	0.71	(0.50, 1.01)	0.83	(0.59, 1.19)
P for trend		0.02		0.47

Crude HR, crude hazard ratio.

Adjusted HR†: multivariable analysis including age, sex, and comorbidities of hypertension, hyperlipidemia, stroke, COPD, CAD, CHF, alcohol-related illness, asthma, obesity, cancer, impaired glucose tolerance, and gestational diabetes, and medication of steroids, diuretics, statins and beta-blockers.

***P < 0.001.

### RNA-seq and differentially expressed genes analysis

**Figure 2:**
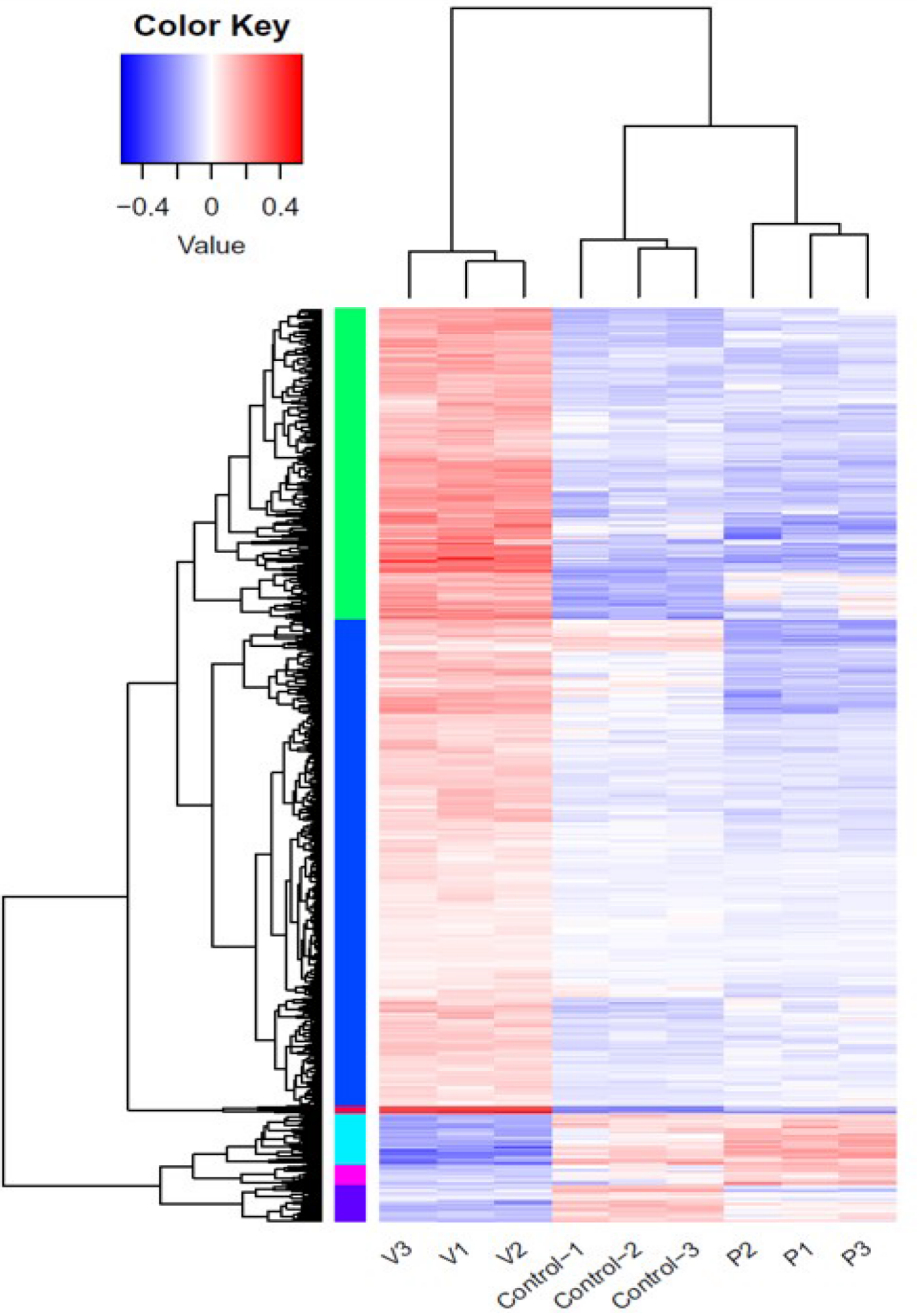
Differential gene expression in control, phenytoin (P)-, and valproate (V)-treated cells. Heatmap of all differentially expressed genes in the control, phenytoin (PHE)-, and valproate (VPA)-treated samples.

The effects of PHE and VPA on the expression of genes associated with T2DM-related pathway were investigated by using control and PHE- and VPA-treated RD cells to construct cDNA libraries for sequencing on an Illumina NovaSeq instrument; three biological replicates were used, and a total of 2.083 × 10^8^ 150 paired-end raw sequencing reads were obtained for all samples. The raw reads were filtered by removing low-quality reads and reads containing N and adaptor sequences, leaving 2.079 × 10^8^ clean reads for downstream bioinformatic analysis. The clean reads were then mapped onto the human GRCh37 genome by using Hisat2 (v2.0.1): 95.28–96.19% were aligned and about 94.42–94.57% were uniquely mapped. The level of gene expression was measured using the read density: the higher the read density, the higher is the level of gene expression. Gene expression was determined by using a formula, which calculates fragments per kilo bases per million reads (FPKM) based on read counts from HT-seq (v0.6.1). There were 57,905 transcripts expressed in the RNA-Seq data. An absolute fold change of ≥ 2 and an adjusted P-value of < 0.05 were used to detect DEGs by using DESeq2 (v1.6.3) package. The heatmap distinguished different transcription profiles between the 3 groups (**Figure 2**).

### Functional analysis of genes regulated by PHE and VPA treatment

**Figure 3:**
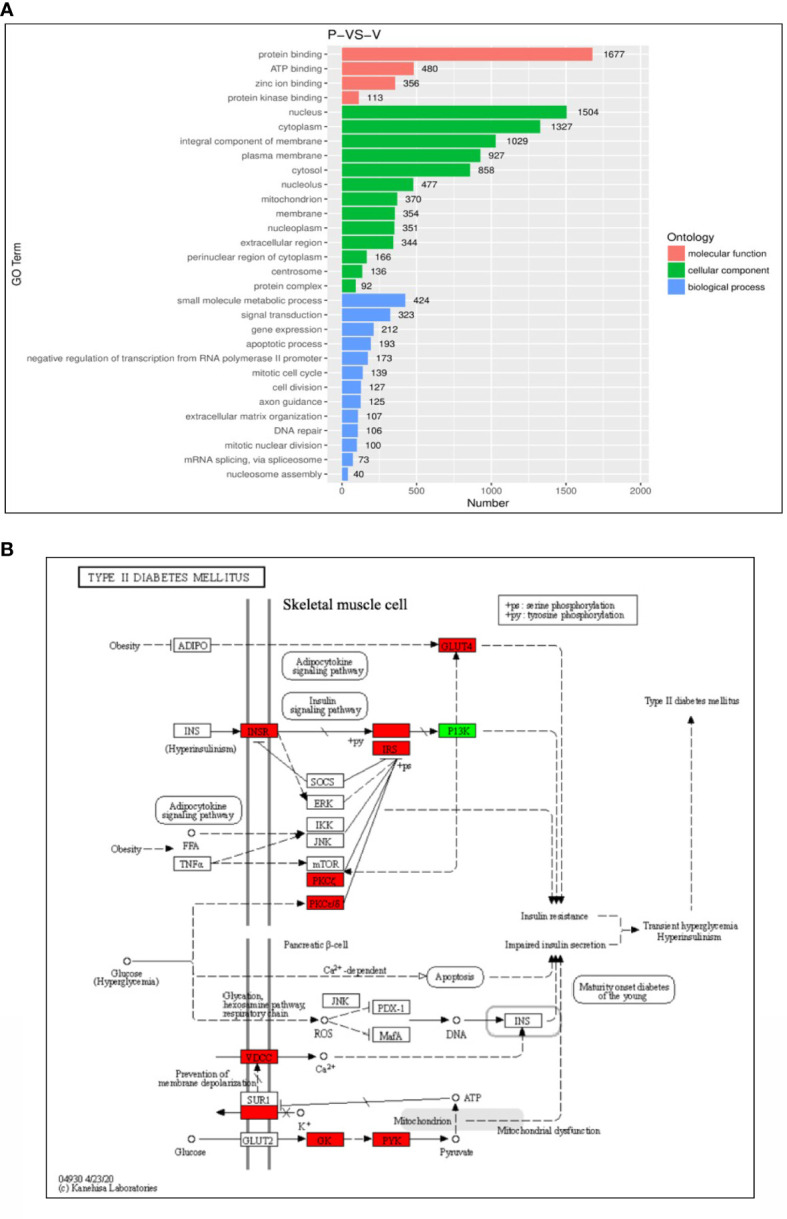
Functional enrichment analysis of differential gene expressions (DEGs). **(A)** Top representatives of gene ontology biological process terms and **(B)** KEGG analysis of T2DM-related DEGs obtained using the samples treated with phenytoin (P) and valproate (V). The red box indicates gene expression: VPA > PHE; the green box indicates gene expression: PHE > VPA, P < 0.05 in **(B)**.

Gene ontology (GO) enrichment analysis was performed to further explore the biological impact of the DEGs induced by PHE and VPA treatments. Several ontologies of GO analysis, such as molecular function, cellular component, and biological process, were obtained. The top biological process terms of the GO analysis associated with up- or down-regulated genes following PHE and VPA treatment, are shown in **Figure 3A**. The figure shows that treatment-induced changes in gene expression were thoroughly different from the perspective of biological functions. The KEGG analysis revealed PHE- and VPA-induced up- and down-regulated DEGs related to T2DM pathway, which are shown in **Figure 3B**. When compared with PHE, VPA increased the gene expression of INSR, VDCC, GK, PYK, PKCζ, PKCε/δ, IRS, and GLUT4 which are beneficial for maintaining glucose homeostasis, while PHE increased only the expression of PI3K in T2DM pathway.

**Figure 4:**
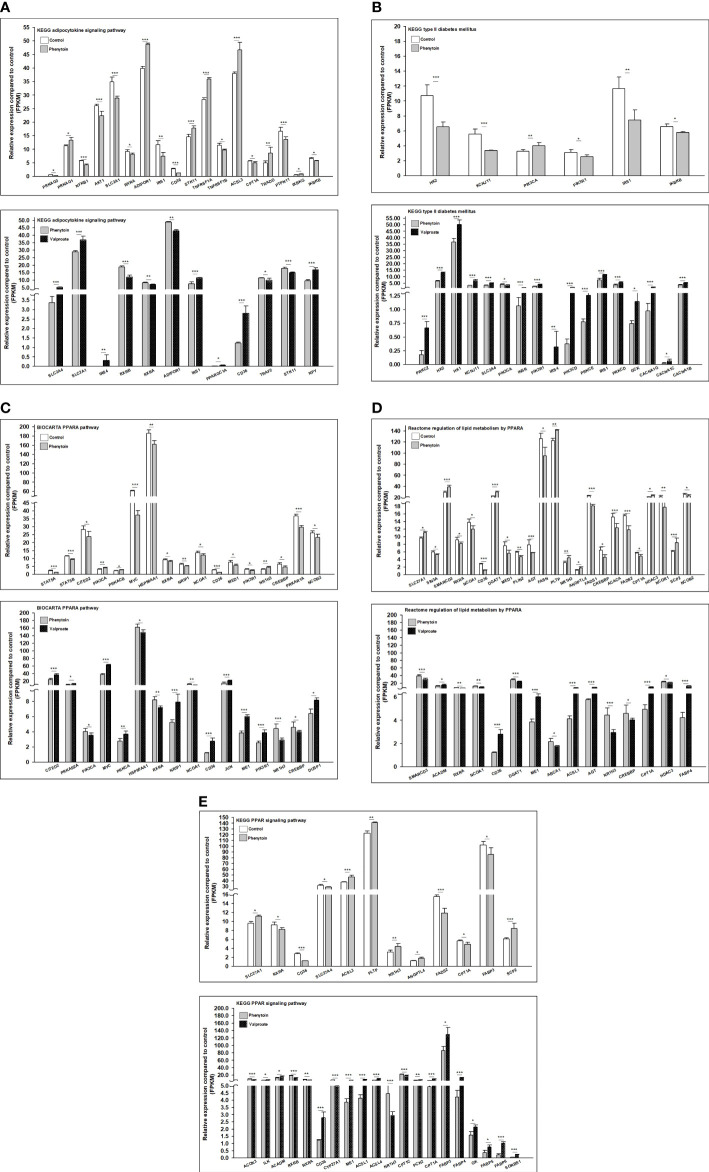
Gene family expression (presented by FPKM) of T2DM-related pathways from RD cells treated with DMSO, PHE, and VPA. Cells were treated with DMSO, PHE (79 μM), and VPA (693.4 μM) for 72 h; mRNA was extracted; and RNA-Seq and DEG analyses were performed. **(A)** KEGG adipocytokine signaling pathway; **(B)** KEGG T2DM pathway; **(C)** BIOCARTA PPARA pathway; **(D)** Reactome regulation of lipid metabolism by PPARα; **(E)** KEGG PPAR signaling pathway. *P < 0.05, **P < 0.01, ***P < 0.001.

The 3 RNA-Seq datasets were analyzed two by two (control vs PHE; VPA vs PHE). We identified the genes associated with the top 10 pathways involved in T2DM, by applying adjP-criterion, as previously published (21); these comparisons are listed in **Table 5**. Compared with the control group, PHE-treated group had a substantially lower level of expression of genes beneficial in glucose homeostasis (59.2% vs. 40.8%, CON vs. PHE); the level of expression of genes beneficial in glucose homeostasis, was substantially higher in VPA- than in PHE-treated group (70% vs. 30%, VPA vs. PHE). Genes associated with 5 T2DM-related pathways, according to Li et al. (21) and those significantly different between CON vs PHE and PHE vs VPA are shown in **Figure 4**.

**Table 5:** Summary of genes significantly different between the 2 groups (CON vs. PHE, left part; VPA vs. PHE, right part).

	Gene numbers significantly different from CON and PHE groups (P < 0.05)	Gene expression (gene numbers that beneficial for blood glucose homeostasis) CON vs. PHE		Gene numbers significantly different from VPA and PHE groups (P < 0.05)	Gene expression (gene numbers that beneficial for blood glucose homeostasis) VPA vs. PHE	
KEGG adipocytokine signaling pathway	18	10	8	12	9	3
KEGG T2DM	6	4	2	17	14	3
BIOCARTA PPARA pathway	17	11	6	16	11	5
Reactome regulation of lipid metabolism by PPARα	23	15	8	15	8	7
KEGG PPAR signaling pathway	12	5	7	20	15	5

### Evaluation of AEDs effect on T2DM-related gene expression and the involvement of PPARγ

**Figure 5:**
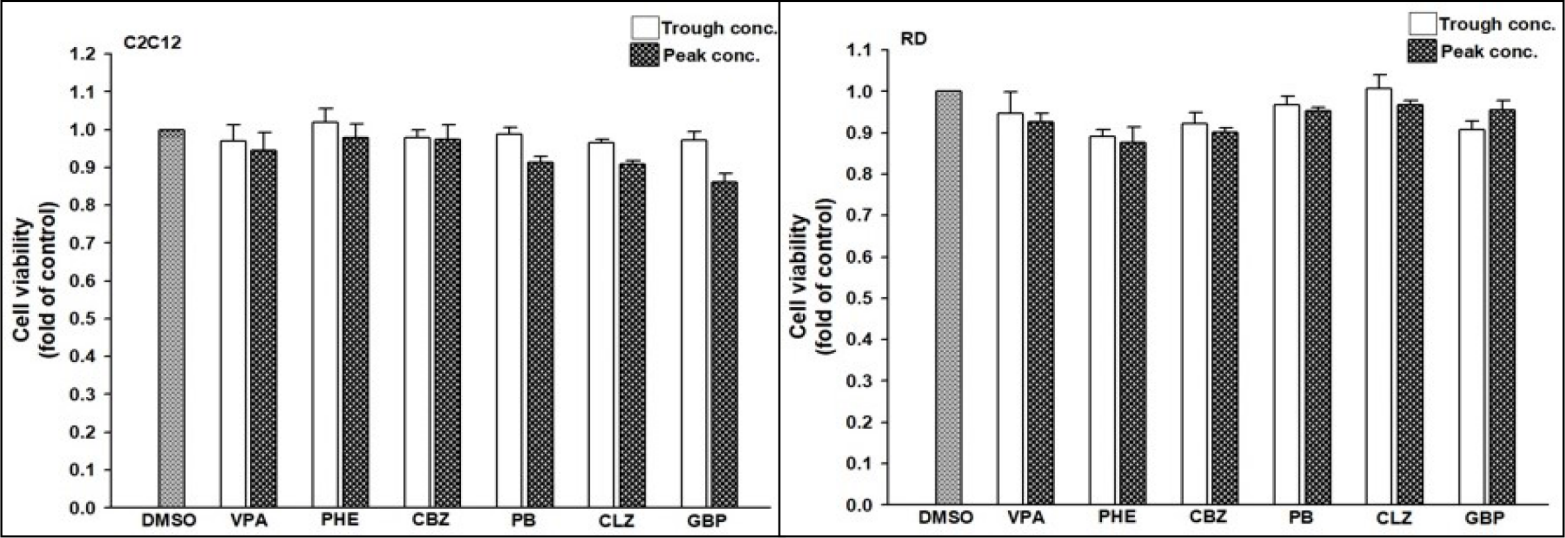
Viability of C2C12 and RD cells after exposure to anti-epileptic drugs (AEDs). (Left) C2C12 and (Right) RD cells were exposed to valproate (VPA, trough: 346.7 μM, peak: 693.4 μM), phenytoin (PHE, trough: 40 μM, peak: 79 μM), carbamazepine (CBZ, trough: 17 μM, peak: 51 μM), phenobarbital (PB, trough: 43 μM, peak: 172 μM), clonazepam (CLZ, trough: 0.06 μM, peak: 0.22 μM), and gabapentin (GBP, trough: 12 μM, peak: 117 μM) for 24 h. Cell viability was monitored using cellular acid phosphatase activity by using 4-nitrophenyl phosphate disodium salt as a substrate. The data are shown as the mean ± SE (error bars; n = 3).

The risk of developing T2DM was lower in patients receiving VPA than in controls. Thus, we intended to perform an in-depth study for verifying the underlying mechanism of action by which AEDs interfere with the risk of developing T2DM. We selected one drug from each category of AEDs to evaluate their effects on cultured cells. Drug concentrations were selected according to the target serum concentrations from trough to peak doses recommended in the Handbook of Pharmacotherapy (35). Since hepatic toxicity of AEDs is well documented (36), we conducted a cell viability assay in 2 rhabdomyosarcoma cell lines: human RD and mouse C2C12 cells. An ACP assay was used to assess cytotoxicity following the exposure of these two cell lines to AEDs for 24 h. Cytotoxicity was not significantly different between the AEDs regardless of the concentration (**Figure 5**).

**Figure 6:**
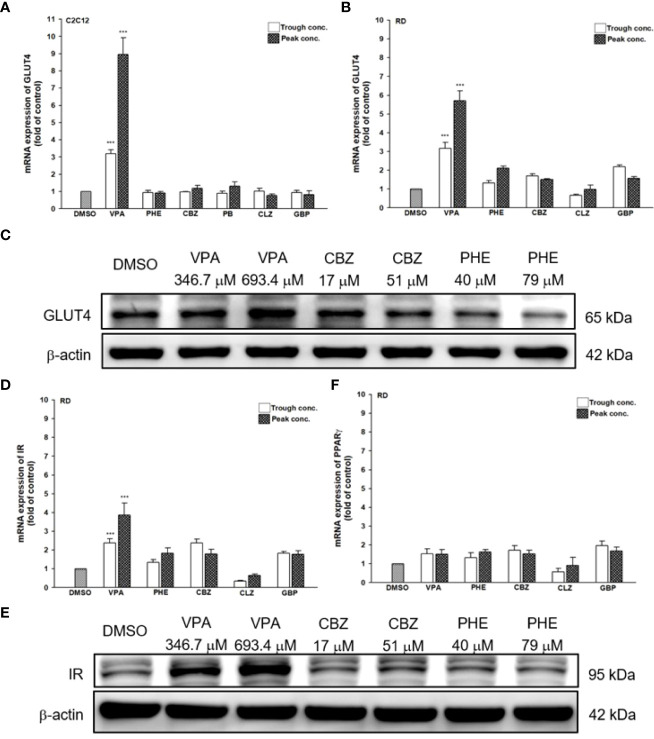
Gene and protein expression of T2DM-related genes after treatment with AEDs. **(A)** C2C12 and **(B, D, F)** RD cells were treated for 24 h with AEDs (each with trough and peak concentrations); after treatments, total RNA was extracted, and the expression levels of GLUT4, IR, and PPARγ and β-actin as a control were analyzed using qRT-PCR. Values were normalized to the expression of β-actin, with the levels of DMSO-treated cells set at 1. Results are expressed as means ± SE (n = 3). ***P < 0.001 compared with control cells as indicated. **(C, E)** RD cells were treated for 24 h with VPA, CBZ, and PHE. Whole-cell extract was harvested, and the expression levels of **(C)** GLUT4 and **(E)** IR and the internal control (β-actin) were analyzed using western blot analysis. A representative blot is shown.

**Figure 7:**
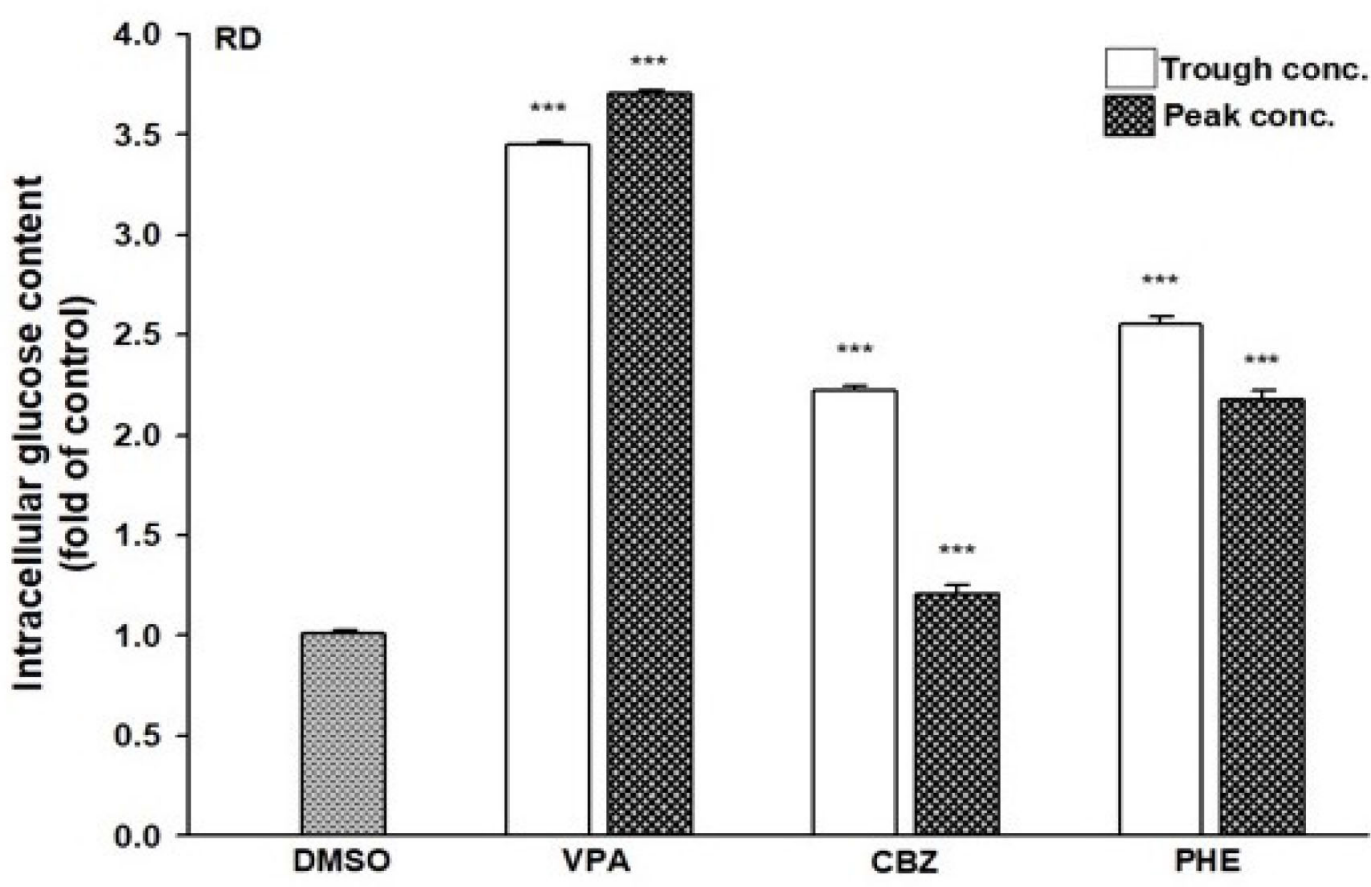
Intracellular glucose content in AEDs-treated cells. RD cells were treated with VPA, CBZ, and PHE for 24 h; cell lysates were extracted; and glucose contents were analyzed using a glucose colorimetric assay kit, according to manufacturer’s instructions. Values were normalized to individual protein contents, with the levels of DMSO-treated cells set at 1. Results are expressed as means ± SE (n = 3). ***P < 0.001 compared with control cells as indicated.

Since AEDs may affect the outcome of T2DM development, we evaluated the effects of AEDs on the expression of GLUT4; as the skeletal muscle is the major site for fuel oxidation (37, 38). In C2C12 cells, VPA significantly induced the expression of GLUT4 up to 3.2- and 8.9-fold after treatment with 346.7 and 693.4 μM VPA, respectively (**Figure 6A**). However, other AEDs did not induce GLUT4 mRNA expression. Treatment of RD cells with 346.7 and 693.4 μM VPA induced GLUT4 mRNA expression up to 3.2- and 5.7-fold, respectively (**Figure 6B**). Increased GLUT4 protein expression was observed in cells treated with VPA, but not in those treated with CBZ and PHE (**Figure 6C**). The interaction between insulin and its target cells leading to the manifestation of insulin-induced effects, relies on IR. Its activation triggers different cellular processes such as glucose uptake and protein synthesis, through an intracellular signaling network (13). We found that treatment with 346.7 and 693.4 μM VPA induced IR mRNA expression up to 2.4- and 3.9-fold, respectively (**Figure 6D**); this effect was also confirmed by assessment of protein expression (**Figure 6E**). However, no significant change in PPARγ mRNA expression was noted after treatment with any AED (**Figure 6F**). Furthermore, we determined the intracellular glucose content in VPA-, CBZ-, and PHE-treated cells by using glucose colorimetric assay; we found that the intracellular content of glucose was higher in VPA-treated cells than in CBZ- and PHE-treated cells. For cells treated with peak concentrations of CBZ and PHE, intracellular glucose was reduced compared with cells treated with trough concentrations (**Figure 7**). These results suggest that, compared with other AEDs, VPA can induce the expression of GLUT4 and IR, and thus enhance glucose uptake and increase intracellular glucose concentration.

### VPA specifically transactivates PPARγ-mediated PPRE promoter activity and mRNA expression of GLUT4

**Figure 8:**
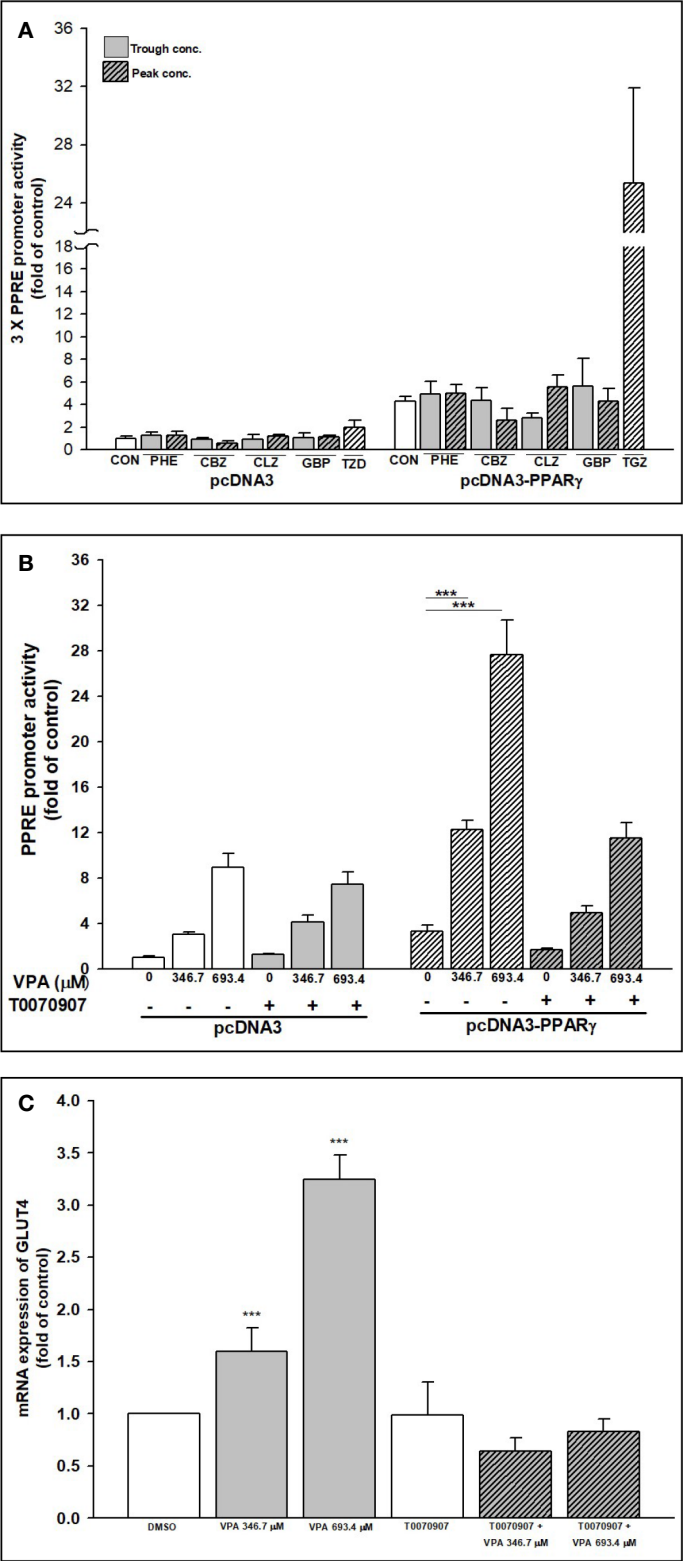
Transactivation of PPARγ–PPRE promoter activity and GLUT4 expression after AEDs treatment. Transient transcriptional assays of 3 × PPRE reporter activity were performed in HepG2 cells to determine the effects of **(A)** AEDs- and **(B)** VPA-, VPA + T0070907-mediated activation of PPARγ. HepG2 cells were co-transfected with a control vector (pcDNA3) or a PPARγ expression plasmid (pcDNA3-PPARγ) and a 3 × PPRE reporter plasmid. Transfected cells were subsequently exposed to AEDs for 24 h. Results are expressed as means ± SE (n = 4), with the levels of DMSO-treated cells set at 1. ***P < 0.001 compared with control cells as indicated. **(C)** RD cells were treated with VPA alone or in combination with T0070907; after treatments, total RNA was extracted, and the expression levels of GLUT4 and β-actin were analyzed using qRT-PCR. Values were normalized to the expression of β-actin, with the levels of DMSO-treated cells set at 1. Results are expressed as means ± SE (n = 3). ***P < 0.001 compared with control cells as indicated.

The ability of AEDs to transactivate PPARγ in a cell-based system was assessed using luciferase assay. All AEDs were tested using trough and peak concentrations. A reporter construct containing 3 × PPRE upstream of a luciferase reporter was transfected into HepG2 cells, which were then treated with AEDs or troglitazone (TGZ, used as a positive control). PPARγ expression was the highest following TGZ treatment (25.4-fold; **Figure 8A**). Even with the present of PPARγ, PHE, CBZ, CLZ, and GBP did not transactivate the PPRE promoter. Only VPA significantly transactivated the PPRE promoter, especially in the presence of PPARγ (12.3- and 27.7-fold increases with 346.7 and 693.4 μM VPA, respectively; **Figure 8B**). The specificity of VPA’s effect was confirmed in the PPARγ reporter assay by blocking the response to VPA with a PPARγ-LBD antagonist, T007. The presence of T007 inhibited the VPA effect in the PPRE reporter assay, in a concentration-dependent manner (**Figure 8B**) and reduced mRNA expression of GLUT4 (**Figure 8C**).

**Figure 9:**
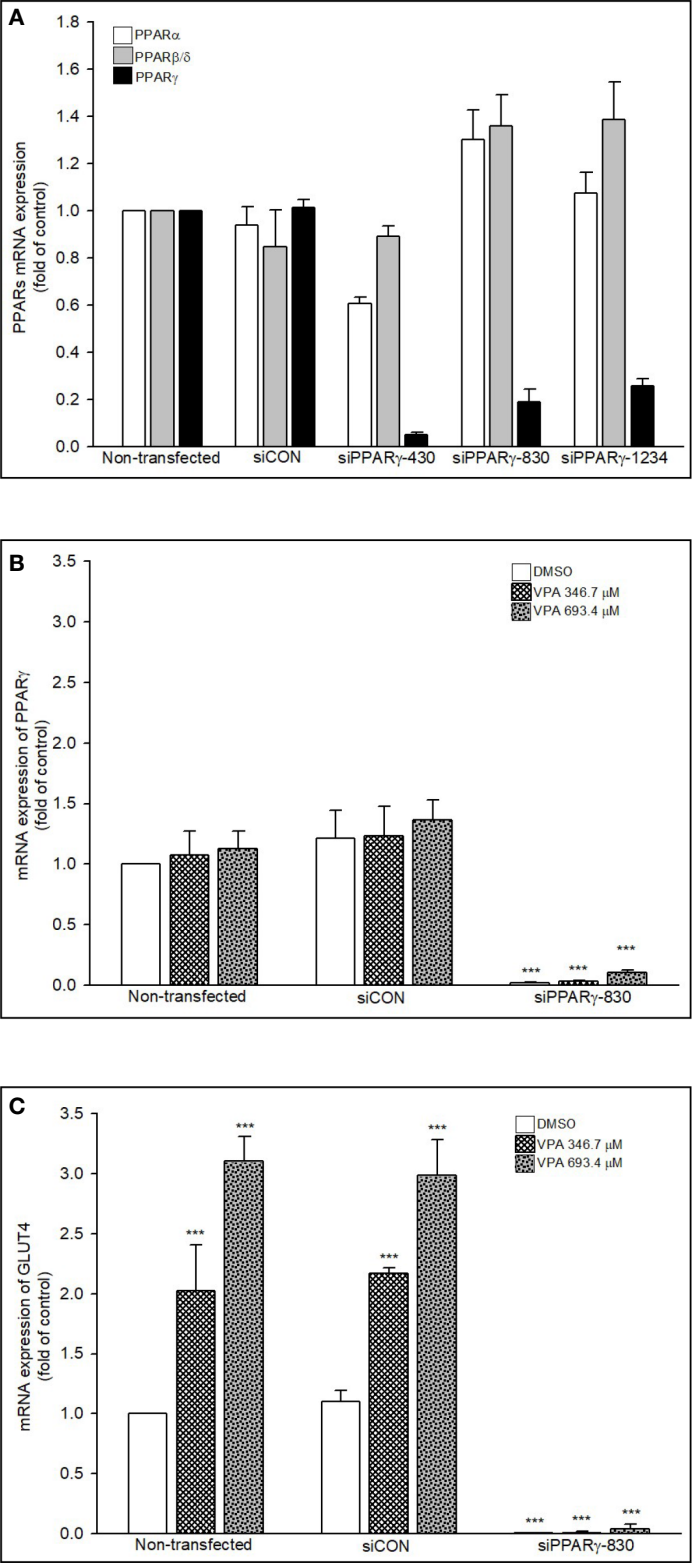
Knockdown of PPARs and mRNA expression of PPARs and GLUT4. **(A, B)** RD cells were transfected with 30 nM siControl or siPPARs for 48 h or treated with **(C)** VPA for additional 24 h. Total RNA was extracted, and the expression levels of PPARs, GLUT4, and β-actin were analyzed using qRT-PCR. Values were normalized to the expression of β-actin, with the levels of DMSO-treated cells set at 1. Results are expressed as means ± SE (n = 3). ***P < 0.001 compared with control cells as indicated.

After we confirmed VPA can specifically induce GLUT4 expression via PPARγ transactivation, we used 3 kinds of PPARγ siRNA (siPPARγ-430, -830, and -1234) to determine whether VPA can also transactivate PPARα or PPARβ/δ. We found that these siPPARγ specifically acted against PPARγ, but not PPARα/β/δ (**Figure 9A**). We selected siPPARγ-830 to investigate the inhibitory effect on the expression of PPARγ and GLUT4 (**Figures 9B, C**). The mRNA expression of PPARγ and GLUT4 was reduced by siPPARγ-830 even in the presence of VPA. These results showed that VPA can specifically induce GLUT4 expression by transactivation of PPARγ signaling pathway.

### Molecular docking

**Figure 10:**
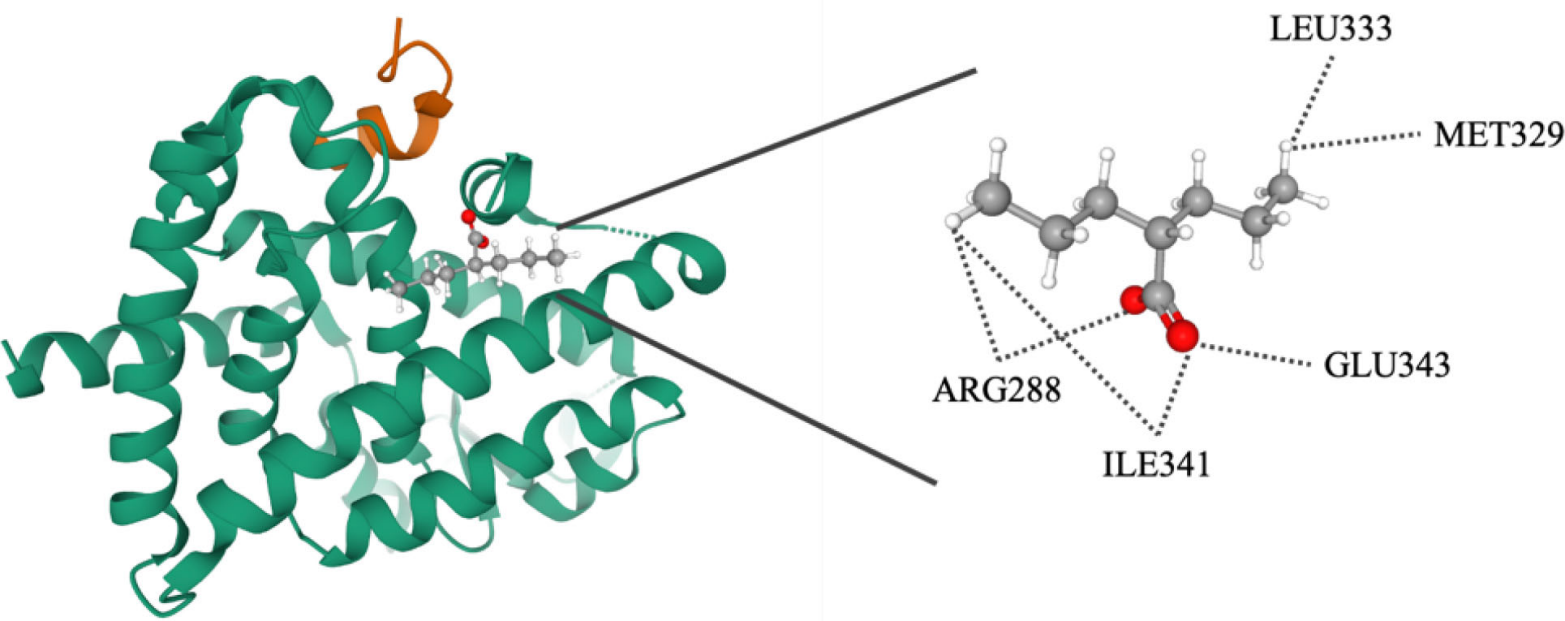
Molecular docking analysis of valproate on PPARγ. Superimposition of docked compounds in the PPARγ-binding pocket of the 3D structure.

Molecular docking was performed to simulate the interactions between PPARγ and its ligand. The ligand of PPARγ was virtually docked to the LBD of PPARγ (PDB entry: 6ILQ) by using the docking program CDOCKER. The docking analysis revealed that the interaction between VPA and PPARγ has an energy score of 32.667. The binding model indicates VPA binds to LBD at residues ARG288, ILE341, GLU343, MET329, and LEU333 (**Figure 10**). Thus, we showed that VPA acts as a partial agonist of PPARγ, providing insight for designing novel PPARγ modulators.

## Discussion

To our knowledge, this is the first population-based study reporting a correlation between epilepsy, AEDs use, and the risk of T2DM, in Asian populations. The risk of T2DM is 1.27 times higher in epileptic patients than in the general population after adjusting for confounding factors and comorbidities (P < 0.001). Furthermore, for patients receiving hydantoin derivatives-based treatment, the risk of T2DM had a 1.26-fold increase when compared with non-epileptic controls. However, even without AEDs treatment, epileptic patients had a higher risk of T2DM than the control cohort (aHR, 1.70; 95% CI, 1.54-1.88, P < 0.001). Furthermore, the risk of T2DM decreased in epileptic patients treated with AEDs when compared to those not treated. Our dose response sub-analysis demonstrated that PHE increased, while VPA decreased the risk of T2DM, in a dose-dependent manner. We also analyzed the T2DM-related pathways interfered by PHE and VPA and the treatment-induced biological disturbances that contribute to T2DM development. In addition, we demonstrated that among AEDs, VPA may act as an effective PPARγ ligand increasing glucose uptake and thus, may be beneficial for epileptic patients by reducing the risk of T2DM development.

T2DM is associated with an increased risk of several neurological disorders, such as dementia, Parkinson’s disease, and multiple sclerosis (11). Data regarding the comorbid association of T2DM and epilepsy exist, however they are sparse. Thus, a population-based study estimating the risk of epilepsy in patients with T2DM, revealed severe hypoglycemia as well as T2DM increased the risk of epilepsy (11). Moreover, a review confirmed the association between T2DM and epilepsy (12). Additionally, epilepsy is often comorbid with obesity (39, 40). These patients are less physically active compared to the general population and thus, they have increased BMI, decreased aerobic strength, poor self-respect, and high levels of anxiety and depression (41). In addition, 61% of the T2DM cases are attributable to obesity, and the risk of T2DM doubles with a weight gain of 5-8 kg (42). In our study, after adjusting comorbidities such as obesity, the correlation between epilepsy and the risk of T2DM still remained significant, suggesting epilepsy may independently increase the risk of T2DM development. Several studies have pointed to the fact that there is an association between epilepsy and T1DM. A meta-analysis evaluated the association between T1DM and epilepsy using data from various sources, including Pubmed, ISI Web of Knowledge, Embase, and Cochrane Library. Of the 13 studies identified, three cohort studies met the inclusion criteria (43). The results showed that T1DM is associated with an increased risk of epilepsy, and this association is also observed in patients younger than 18 years old. The findings indicate that individuals with T1DM have a significantly higher risk of developing epilepsy compared to those without the condition. A review examines the potential relationship between epilepsy and T1DM and investigates the underlying mechanisms. The presence of anti-glutamic acid decarboxylase antibodies (GAD-Abs), which are associated with T1DM and various neurological disorders, including epilepsy, may play a role in this link (44). Additionally, metabolic conditions such as hypoglycemia and hyperglycemia, which are common in diabetic patients, may also be implicated. However, the exact pathogenetic mechanisms behind this association remain unclear.

In epileptic patients, T2DM might develop because of mitochondrial dysfunction, adiponectin deficiency, or obesity, these three mechanisms might sequentially or concurrently induce the biological drivers of T2DM in epileptic patients (45). Drug-induced diabetes is a clinical condition reported by physicians, although the underlying mechanisms of the diabetogenic effects are unknown: alteration of insulin secretion and sensitivity, direct cytotoxic effects on pancreatic cells, and increase in glucose production may be involved (16). Furthermore, drug-induced diabetes is becoming a global issue because its potential to induce severe CVD complications. A study in Taiwan using the NHI reference database revealed that 71% of epileptic patients used AED monotherapy (46). Approximately one third of epileptics take two or more AEDs. CBZ, PHE, and VPA are the most frequently used drugs in monotherapy, and a combination of CBZ and VPA is used in polytherapy (46). PHE induces hyperglycemia particularly at toxic concentrations, secondary to the inhibition of insulin release and a subsequent post-binding defect in insulin action (16). Patients with PHE-induced hyperglycemia have an increased insulin requirement which suggests the occurrence of insulin resistance. However, the reduction of PHE dose resulted in improvement of hyperglycemia. *In vitro* studies assessing the effect of PHE on both insulin receptor binding and post-binding function in a primary culture system of adipocytes showed a 57% reduction in maximum [^14^C]3-0-methylglucose transport in the presence of PHE and no effect on maximum insulin binding. Therefore, PHE administration can result in insulin resistance by induction of a post-binding defect in insulin action. VPA treatment may increase the risk of insulin resistance and consequently of T2DM (47–49). However, the studies investigating these effects have small dimensions. Further efforts are needed to explore the relationship between AEDs use and occurrence of diabetes. Our cohort study demonstrated epilepsy increased the risk of T2DM development. However, in patients receiving AEDs treatment, this risk decreased. Moreover, increasing PHE dosage increased the risk of T2DM, while increasing the DDD of VPA decreased the risk. Thus, clinicians should be aware that both medication and the disease itself may alter glycemic status. Patients receiving drugs known to alter blood glucose levels should be identified and closely monitored.

We further investigated the mechanisms underlying these effects determined by PHE and VPA. Skeletal muscle is the primary target of various factors, for increasing insulin-stimulated glucose uptake, utilization, and disposal (14). RD cells preferentially use glycolytic over oxidative metabolism (50). They exhibit a low but significant insulin-stimulated glucose uptake, in the range of 20–25%. Thus, we chose RD cells to determine the mechanisms of the diabetogenic effect of PHE/VPA treatment. After drug treatment, gene expression was evaluated in triplicate. We used high throughput RNA sequencing (RNA-seq) for its unbiased ability to detect expressed genes with greater sensitivity and accuracy than gene expression microarrays. With appropriate bioinformatics tools, regulatory events affecting genes and associated pathways can be identified more efficiently than in single-gene assays. These tools were applied to identify genes and pathways modified by PHE or VPA in RD cells, when compared to the corresponding not treated controls. To understand the effect of PHE and VPA on the gene expression of the whole muscle genome, RNA-seq was performed on RD cells cultured for 72 hrs in triplicate. After read alignment and gene expression quantification, differential expression analysis of genes and pathways was undertaken. After analysis, the possible candidate genes with significant response (P < 0.05) and those corelated with T2DM (21) were selected and compared between the PHE and VPA groups. We observed that, control, PHE, and VPA treated samples clustered into distinct groups. Statistical analysis showed that PHE and VPA treatment induced differential expression of genes in RD cells, with FDR ≤ 0.05, the top differentially expressed genes ranked by significance are shown in heatmap form. We identified genes that had significantly different levels of expression among treated group. These gene sets included KEGG adipocytokine signaling pathway, KEGG T2DM, BIOCARTA PPARA pathway, reactome regulation of lipid metabolism by PPARα, and KEGG PPAR signaling pathway. These findings show an important effect of these AEDs on RD cells. The number of genes beneficial for glucose homeostasis were substantially higher in control vs. PHE, and VPA vs. PHE (59.2% vs. 40.8%, 70% vs. 30%, respectively). VPA increased the expression of GLUT4, IR, and thus intracellular glucose content. We hypothesized that VPA might alter the levels of expression of these genes by PPARγ transactivation and VPA induced GLUT4 expression by PPARγ transactivation.

PPARγ, the molecular target of the TZDs, modulates multiple pathways involved in insulin sensitivity. It represents a promising target for drugs against pathologies such as T2DM and metabolic syndrome (2). Both TZDs, rosiglitazone and pioglitazone, are still available in many countries for the T2DM control (51), although the European Medicines Agency and U.S. Food and Drug Administration issued various warnings on their CVD safety issues. In patients with T2DM, when compared with placebo or active controls, rosiglitazone increases significantly the risk of heart failure, and to some extent that of myocardial infarction, but not the risk of stroke, and it does not influence the cardiovascular mortality and all-cause mortality (52). Compared to rosiglitazone, pioglitazone exerts beneficial effects on the plasma lipid profile, leading to a lower risk of acute myocardial infarction, stroke, or heart failure (51). However, the clinical use of pioglitazone is also limited by the occurrence of several adverse events, including body-weight gain, fluid retention, and possibly bladder cancer (53). Thus, it needs to develop safer PPARγ to reduce the abovementioned adverse reactions.

Skeletal muscle is the main tissue responsible of glucose uptake, and various factors can stimulate this process and increase glucose utilization (37). In contrast, muscle contraction increases insulin-mediated glucose disposal. GLUT4 levels in skeletal muscle are adjusted by transcriptional regulation, and overexpression of GLUT4 in skeletal muscle increases insulin- and contraction-stimulated glucose uptake and metabolism (54). Impaired insulin stimulation of glucose uptake in adipose tissue and skeletal muscle is one of the earliest defects detected in insulin-resistant states (55). In these states, the level of expression of GLUT4 is substantially lower in adipose tissue, as reported by preclinical and clinical studies, however it is not modified in muscle tissue, which indicates a tissue-specific nature of GLUT4 gene regulation in pathological and physiological states (55). GLUT4 density in muscle fibers from DM patients was 9% lower than in those from weight-matched non-diabetic obese subjects and 18% lower than in those from the lean control group (56). Thus, decrease of GLUT4 expression leads to insulin resistance and consequently to T2DM. Increased muscle GLUT4 expression improves insulin action and glucose disposal and enhances muscle glycogen storage (38). The regulation of GLUT4 gene possesses interest from a clinical point of view, because insulin-mediated glucose homeostasis depends on GLUT4 protein levels in muscles (56). PPARγ binds to cis elements on the GLUT4 promoter and keeps it in a repressed state; TZDs binding to PPARγ causes the detachment of corepressors and the attachment of coactivators, which subsequently leads to increased GLUT4 expression and enhanced insulin responsiveness (37). There are reports of VPA reducing the blood glucose level and fat deposition in adipose tissue and liver of mice and rats (57, 58). In addition, VPA treatment determines lower fasting plasma glucose levels (59), and can be safely prescribed, not causing insulin resistance and its associated complications (60). VPA-treated patients presented lower blood glucose levels compared with controls (59, 61–63). However, VPA induced weight gain in several clinical studies (64–66), although the exact mechanisms remain unknown. Recently, the possibility of repurposing VPA for the treatment of different diseases was investigated (67–69). Here, we demonstrate for the first time that VPA may be repurposed for the treatment of T2DM as it specifically transactivates PPARγ. Furthermore, we demonstrated VPA as a PPARγ agonist may reduce the risk of TD2M in epileptic patients. Further research is needed to confirm these results.

The PPARγ protein comprises an N-terminal regulatory domain, a central DNA-binding domain, and a C-terminal LBD (amino acids 204-477). The LBD consists of 13 α-helices and a small four-stranded β-sheet. Helix H12 of the ligand-dependent activation domain (AF-2), is essential for ligand binding and PPAR activation (2). The large size and the flexibility of the binding pocket allow PPARγ to interact with structurally distinct ligands. The binding pose indicates VPA interacts with the LBD at 5 residues. Thus, we showed that VPA could act as a new partial PPARγ agonist, providing insight for the development of novel PPARγ modulators.

Our study had several limitations. First, the database we used may have misclassified epilepsy and T2DM, limiting the reliability and validity of the study. However, in Taiwan, universal health insurance is distributed, and as a result, specialists enact a peer review system, to reduce the possibilities of bias. Second, some factors associated with T2DM are unmeasured or poorly captured in claims data (e.g. smoking habits, alcohol intake, body mass index, lifestyle and dietary habits, and family history of T2DM, are not available in NHIRD database). However, they are unlikely to be associated with the choice of epilepsy treatment. Therefore, the magnitude of their impact is negligeable and they would likely not influence our findings. Third, this claims database was initially created for charging purposes, and as some information was anonymized, we could not get any individual information directly from the patients. Fourth, exposure, outcome, or covariate misclassification cannot be excluded, as this claims database does not include clinical laboratory data such as blood glucose and HbA1c levels, and thus, we could not assess the severity of T2DM. Exposure was evaluated based on medication dispensing. In this study, both epilepsy and T2DM were accurately diagnosed and coded by specialists according to standard symptomatic criteria, research facility information, and imaging findings. To minimize outcome misclassification, we used validated, age-specific definitions of T2DM with high positive predictive values in health care databases. Additionally, this study reduced the confounding effect of medication by adjusting for comorbidities. However, more information should be obtained from other databases to conduct a comprehensive prospective study or randomized controlled trial to further investigate the relationship between epilepsy, AEDs, and T2DM. Most important, evidence derived from a retrospective cohort study is typically lower in statistical quality than that derived from clinical trials, because of numerous sources of inherent bias, including the classification bias. However, the NHI program has a high coverage rate, and medical reimbursement specialists and peer reviewers scrutinize all insurance claims, ensuring that the diagnoses and coding of diseases are highly reliable. The nondifferential misclassification allows that our results are not invalidated by the classification bias.

This study had several strengths. First, we used a nationwide, population-based cohort of patients with anonymized data to minimize selection bias. Additionally, we evaluated the effect of epilepsy and AEDs on the risk of development of T2DM over a prolonged follow-up period. Moreover, by adjusting for age and sex in a 1:1 ratio, we accounted for confounders that may affect the occurrence of T2DM. Finally, although detection bias may have occurred if patients had more hospital visits than the control population by increasing the possibility of detecting T2DM, the risk of T2DM was still increased 1.27-fold in patients with ≥ 2 hospital visits per year. Overall, this is the first study to investigate the epilepsy-associated risk for developing T2DM and evaluate the effects of AEDs on T2DM-related pathway gene expression and on PPARγ transactivation.

## Conclusion

On the basis of our cohort study and NGS database research, we found that epilepsy is associated with an increased risk of developing T2DM. Treatment with AEDs decreased this risk when compared to no treatment. However, increased DDD of PHE, but not of VPA, increased risk of T2DM when compared to non-diabetic population. These findings highlight that the initial choice of one AED could impact the incidence of T2DM. Patients and clinicians concerned about the potential metabolic adverse effects of the AEDs treatment should consider initiating therapy with VPA, which possesses the lowest risk of T2DM. We suggest a novel paradigm to increase GLUT4 expression in skeletal muscle cells, by using an AED used in clinical practice that also has regulatory effects on PPARγ. We believe that further unveiling the mechanisms that regulate GLUT4 gene expression in diabetes will hopefully result in finding effective ways to improve overall insulin sensitivity in epileptic populations. We demonstrated for the first time that PHE and VPA administration have different effects on the level of expression of genes related to T2DM-associated pathway. We demonstrated that VPA could induce expression of GLUT4 via the specific transactivation of PPARγ, and thus increase glucose uptake in muscle cells. Furthermore, it also increases the expression of IR. Future preclinical studies will clarify whether VPA can mitigate the risk of T2DM. In this context, repurposing VPA provides a time- and cost-effective alternative for preventing the occurrence of T2DM in patients with epilepsy.

## Data availability statement

The original contributions presented in the study are included in the article/supplementary material. Further inquiries can be directed to the corresponding authors.

## Ethics statement

The studies involving human participants were reviewed and approved by Research Ethics Committee of China Medical University and Hospital in Taiwan. Written informed consent for participation was not required for this study in accordance with the national legislation and the institutional requirements.

## Author contributions

Conceptualization, NT, TYW, YJF, and YPL. Methodology, NT, TYW, YJF, and YPL. Software, CLL, FYC, CCNW, CYH, and FJT. Validation, TYW, and YPL. Formal analysis, CLL, FYC, CCNW, and CYH. Investigation, NT, TYW, YJF, and YPL. Resources, CLL, CCNW, CYH, FJT, and YPL. Data curation, CLL, FYC, CCNW, and YPL. Writing-original draft preparation, all authors. Writing-review and editing, all authors. Visualization, all authors. Supervision, NT, TYW, YJF, and YPL. Project administration, FJT, and YPL. Funding acquisition, NT, TYW, YJF, and YPL. All authors contributed to the article and approved the submitted version.

## Glossary

**Table:**

αMEM	Minimum Essential Medium alpha
ACP	Acid phosphatase
adjP	Adjusted P
AEDs	Anti-epileptic drugs
AF	Atrial fibrillation
aHR	Adjusted hazards ratio
ANOVA	One-way analysis of variance
BMI	Body mass index
CAD	Coronary artery disease
CBZ	Carbamazepine
CHF	Congestive heart failure
CIs	Confidence intervals
CLZ	Clonazepam
COPD	Chronic obstructive pulmonary disease
CVD	Cerebrovascular disease
DDD	Defined daily dose
DEGs	Differentially expressed genes
DM	Diabetes mellitus
DMEM	Dulbecco's modified Eagle's medium
DPP-4	Dipeptidyl peptidase-4
FA	Fatty acid
GBP	Gabapentin
GK	Glycerol kinase
GLUT4	Glucose transporter type 4
GO	Gene Ontology
HCS	HiSeq Control Software
HRs	Hazard ratios
ICD-9-CM & ICD-10-CM	International Classification of Diseases Ninth & Tenth Revision Clinical Modification
IR	Insulin receptor
IRS	Insulin receptor substrate
KEGG	Kyoto Encyclopedia of Genes and Genomes
LBD	Ligand-binding domain
LDL	Low-density lipoprotein
LGTD	Longitudinal Generation Tracking Database
MHW	Ministry of Health and Welfare
MSigDB	Molecular Signatures Database
NGS	Next-generation sequencing
NHI	National Health Institute
NHIRD	National Health Insurance Research Database
NR	Nuclear receptor
PB	Phenobarbital
PDB	Protein Data Bank
PE	Paired-end
PHE	Phenytoin
PI3K	Phosphoinositide 3-kinases
PKC	Protein kinase C
PPAR	Peroxisome proliferator-activated receptor
PPRE	Peroxisome proliferator response element
PYK	Pyruvate kinase
RXR	Retinoid X receptor
SE	Standard error
SGLT	Sodium-glucose cotransporter
siRNAs	Small interfering RNAs
T007	T0070907
T2DM	Type 2 diabetes mellitus
TZDs	Thiazolidinediones
VDCC	Voltage-gated calcium channels
VPA	Valproate
